# *FCN3* functions as a tumor suppressor of lung adenocarcinoma through induction of endoplasmic reticulum stress

**DOI:** 10.1038/s41419-021-03675-y

**Published:** 2021-04-15

**Authors:** Haeyeon Jang, Yukyung Jun, Suyeon Kim, Eunjeong Kim, Yeonjoo Jung, Byung Jo Park, Jinseon Lee, Jhingook Kim, Sanghyuk Lee, Jaesang Kim

**Affiliations:** 1 Department of Life Science, Ewha Womans University, Seoul, Republic of Korea; 2 Ewha Research Center for Systems Biology, Ewha Womans University, Seoul, Republic of Korea; 3 Ewha-JAX Cancer Immunotherapy Research Center, Seoul, Republic of Korea; 4 Center for Supercomputing Applications, Division of National Supercomputing, Korea Institute of Science and Technology Information, Daejeon, Republic of Korea; 5 Department of Thoracic and Cardiovascular Surgery, Samsung Medical Center, Seoul, Republic of Korea; 6 Samsung Biomedical Research Institute, Samsung Medical Center, Sungkyunkwan University School of Medicine, Seoul, Republic of Korea

**Keywords:** Non-small-cell lung cancer, Apoptosis, Endoplasmic reticulum

## Abstract

In this study, we report a novel function of *FCN3* (Ficolin 3), a secreted lectin capable of activating the complement pathway, as a tumor suppressor of lung adenocarcinoma (LUAD). First, the expression of *FCN3* was strongly down-regulated in cancer tissues compared to matched normal lung tissues, and down-regulation of *FCN3* was shown to be significantly correlated with increased mortality among LUAD patients. Interestingly, while ectopic expression of *FCN3* led to cell cycle arrest and apoptosis in A549 and H23 cells derived from LUAD, the secreted form of the protein had no effect on the cells. Rather, we found evidence indicating that activation of the unfolded protein response from endoplasmic reticulum (ER) stress is induced by ectopic expression of *FCN3*. Consistently, inhibition of ER stress response led to enhanced survival of the LUAD cells. Of note, the fibrinogen domain, which is not secreted, turned out to be both necessary and sufficient for induction of apoptosis when localized to ER, consistent with our proposed mechanism. Collectively, our data indicate that *FCN3* is a tumor suppressor gene functioning through induction of ER stress.

## Introduction

Large-scale sequencing projects utilizing massively parallel sequencing techniques led to extensive molecular profiling of diverse types of cancer^1,2^. Included is the lung cancer which has been one of the leading causes of cancer-related death for over 25 years^3^. However, understanding of carcinogenesis based on functional dissection of numerous somatic mutations and differentially expressed genes (DEGs) is still very much limited. Most importantly, it is necessary to distinguish the so-called driver and passenger genes which in turn requires functional analyses and experimental validations. This applies to tumor suppressors as well as oncogenes given that presumptive loss-of-function mutations bearing nonsense mutations and DEGs with decreased expression in tumor tissues are found in large numbers.

*FCN3* was originally identified as the Hakata Antigen, a macroglycoprotein which reacted with the sera of systemic lupus erythematosus patients^4^. Analysis of the cDNA sequence showed that the encoded protein contained an N-terminal collagen-like domain and a C-terminal fibrinogen-like domain. This structural feature led to defining the ficolin/opsonin p35 gene family consisting of *FCN1*, *FCN2*, and *FCN3* and their function in complement activation^5–7^. The collagen-like domain mediates oligomerization, and the fibrinogen-like domain binds to N-acetyl compounds, frequently found on the surface of various microorganisms. They circulate in association with Mannose-binding lectin-associated serine proteases, and binding to targets converts the proteases into active forms which in turn results in complement activation via the lectin pathway.

*FCN3* is highly expressed in the lung and liver, the latter presumably accounting for the circulating portion of the protein^8^. Low serum levels of FCN3 are associated with susceptibility to fever and neutropenia suggesting a role in innate immunity^9^. Consistently, a patient with homozygous mutation in *FCN3* was reported to have suffered from recurrent infections^10^. FCN3 also binds to apoptotic cells which promotes adhesion and uptake by macrophages^11^. In contrast to serum FCN3, the function of the protein generated by ciliated bronchial epithelial cells and type II alveolar epithelial cells in the lung is unknown although secretion into bronchus and alveolus suggests a role in fighting infection in the airway as well^8^.

Reports on the association between *FCN3* with cancer are limited to its potential utility as a marker. Specifically, increased serum levels of FCN3 protein have been described for acute myeloid leukemia and ovarian cancer^12–14^. In contrast, Li and Lin reported that for Chinese patients with esophageal cancer, the elevated level of FCN3 protein was shown to be associated with longer overall survival and disease-specific survival^15^. Here, we present data for the first time indicating that *FCN3* is a tumor suppressor gene of LUAD. Interestingly, the secreted extracellular FCN3 protein showed no effect in regulating the cell cycle or apoptotic process. Rather, the ER stress induced by intra-ER FCN3 appears to account for its role as a tumor suppressor.

## Materials and methods

### Patient samples and transcriptome analyses

Tumor and normal tissue samples were obtained from patients who had undergone curative surgery at the Samsung Medical Center (Korea). RNA Sequencing (RNA-seq) for the said samples has been described and the RNA-seq data were processed following the TCGA pipeline of MapSplice-RSEM^16,17^ using Ensembl GRCh37 (release 81) genome and transcript models. DEGs were obtained using Voom^18^ with the false discovery rate (FDR) < 0.001. TCGA gene expression data were downloaded at level 3 from the Broad GDAC Firehose website (released on January 28, 2016)^19,20^.

### Cell culture and reagents

Human non-small cell lung cancer cell lines A549 and NCI-H23 were purchased from the American Type Culture Collection (Manassas, VA). Cells were typically cultured in RPMI-1640 (WELGENE, Korea) supplemented with 10% fetal bovine serum (Gibco, Carlsbad, CA) and 1% penicillin/streptomycin (Gibco) in humidified incubators at 37°C with 5% CO_2_. Recombinant FCN3 was obtained from R&D systems (Minneapolis, MN). Sodium 4-phenylbutyrate (4-PBA) was purchased from Sigma Aldrich (St Louis, MO). Cells were confirmed negative for mycoplasma contamination.

### Ectopic expression of *FCN3* and derivatives

The full-length coding sequence (CDS) region of human *FCN3* (*FCN3* WT) was amplified by RT-PCR using Human Universal Reference Total RNA purchased from Clontech Laboratories (Mountain View, CA). The collagen-like domain (*FCN3* 1-90) and the fibrinogen-like domain (FBG) were PCR-amplified from *FCN3* CDS. For the FBG domain with the signal peptide at the N-terminus end (SP + FBG), the signal peptide sequence at the N-terminus of *FCN3* was attached to the FBG domain by overhang PCR technique. Each of the DNA fragments was tagged with V5 epitope at the C-terminus and ligated into pLZRS-IRES-GFP vector. Virus expressing only GFP was used as the control. Preparation of high titer virus was carried out using 293GPG cells following previously published protocols with minor modifications^21^. Details on the sequences of oligonucleotide primers and production of pseudotyped viral particles are available upon request. Cells were transduced with each virus in the presence of 4 µg/ml of polybrene (Sigma Aldrich).

### RT-PCR

Total RNA from lung tissues was extracted with TRI Reagent (Ambion, Austin, TX) and reverse transcribed using ImProm-II™ reverse transcriptase (Promega, Madision, WI) to synthesize cDNA. After amplification with Platinum-taq DNA polymerase (Invitrogen, Carlsbad, CA), FCN3 expression was analyzed by gel electrophoresis. For real-time RT-PCR analysis, total RNA was extracted using miRNeasy Mini kit (Qiagen, Valencia, CA) according to the manufacturer’s protocol. cDNA was synthesized from 1 µg of total RNA using ImProm-II™ reverse transcriptase (Promega, Madison, WI). Approximately 5 ng of cDNA was subjected to PCR amplification using SYBR Select Master Mix (Applied Biosystems by Life Technologies, Austin, TX) on a CFX96 Real-time PCR detection system (Bio-Rad, Hercules, CA). *ACTB* and *HPRT1* were used as dual internal reference genes. Oligonucleotide primers used for RT-PCR are listed in Supplementary Table 1.

### Immunofluorescence assay

Cells were seeded on 4-well slides and transduced with retrovirus. 2 days after transduction, cells were fixed with 4% paraformaldehyde, permeabilized with 1% Tween-20 and blocked with 1% BSA. The cells were incubated with the primary antibody against V5 (Invitrogen) overnight at 4 °C followed by incubation with biotinylated goat anti-mouse IgG antibody (H + L) (Vector Laboratories, BA-9200). Cells were subsequently labeled with AMCA using Fluorescent Streptavidin Kit (Vector Laboratories; AS-1200). For endoplasmic reticulum staining, ER Staining Kit-Red Fluorescence-Cytopainter (Abcam, Cambridge, MA; ab139482) was applied according to the manufacturer’s instruction. The slides were mounted using ProLong Gold Antifade Reagent (Invitrogen), and the cells were examined by LSM 880 confocal laser scanning microscope (Carl Zeiss, Germany).

### Immunoblotting assay

Cells and tissues were lysed with cold RIPA buffer (50 mM Tris-Cl, PH 8.0, 2 mM EDTA, 150 mM NaCl, 1% NP-40, 0.5% Na-Deoxycholate, 2% SDS, 10 mM NaF) supplemented with mixture of protease inhibitors (Sigma) and phosphatase inhibitors (Sigma). For the tissue samples, sonication was additionally applied. The concentration of total protein was measured using BCA protein assay kit (Thermo Scientific Pierce, Rockford, IL). For the soluble protein detection, the conditioned media was collected and centrifuged at 13,000 rpm for 20 min before collecting the supernatant. Proteins were resolved by SDS PAGE and electrotransferred to PVDF membrane. Primary antibodies used in immunoblot analyses are as follows: anti-FCN3 (R&D systems; AF-2367), anti-V5 (Invitrogen; R96025), anti-p53 (Santa Cruz Biotechnology, Santa Cruz, CA; sc-126), anti-p21 Waf1/Cip1 (Cell Signaling Technology, Beverly, MA; 2947), anti-CDC25C (Cell Signaling Technology; 4688), anti-Cyclin B1 (Cell Signaling Technology; 4138), anti-Cyclin D1 (Cell Signaling Technology; 2926) anti-PARP (Cell signaling Technology; 9542), anti-HSPA5(Abcam; ab21685), anti-DDIT3 (Cell Signaling Technology; 2895), and anti-α-Tubulin (Sigma; SAB3501072). After applying appropriate secondary antibodies, proteins were detected by enhanced chemiluminescence detection kit (AbClon, Korea) and ChemiDoc™ Imaging system (Bio-Rad)

### Colony formation assay

Two days after retroviral transduction, 1 × 10^3^ A549 cells and 2 × 10^3^ H23 cells were replated in 6-well plate in duplicates. A549 and H23 cells were stained after 8 and 12 days, respectively, with 0.1% Coomassie Blue in 45% methanol and 10% acetic acid solution. The colonies were counted using Gel Doc XR system (Bio-Rad) with Quantity One^®^ 1-D analysis software (Bio-Rad).

### BrdU immunofluorescence assay

Cells were grown on 4-well slides and transduced with retrovirus. After 3 days, BrdU (Sigma Aldrich) was applied at 3 µg/ml for 3 h in dark. Cells were washed with PBS, and fixed with 4% paraformaldehyde for 30 min and treated with 2N HCl for 30 min. The cells were blocked with 5% donkey serum with 0.2% Triton X-100 for 1 h followed by incubation with anti-BrdU antibody (Abcam; ab1893) for 2 h. Alexa-594 donkey anti-sheep antibody (Invitrogen) was used as the secondary antibody, and cells were counterstained with 4’,6-diamidino-2-phenylindole (DAPI). The immunofluorescent images were acquired by Axiovert 200 microscope (Carl Zeiss, Germany).

### Flow cytometry

For cell cycle analysis, a total of 5×10^5^ cells were collected and fixed with 70% ethanol overnight. Cells were subsequently stained in dark at room temperature for 15 min with PBS buffer containing 25 µg/ml 7-amino-actinomycin D (BD Biosciences, San Jose, CA) and 10 µg/ml RNase A (Sigma Aldrich) and 0.1% Triton X-100 prior to flow cytometric analysis. For detection of apoptotic cells, cells were washed with cold PBS and re-suspended in 1x Annexin V binding buffer (BD Biosciences) at a concentration of 1×10^6^ cells/ml. Annexin V-V450 (BD Biosciences) and 7-aad (BD Biosciences) were used according to the manufacturer’s instruction. For GFP-expressing cell population analysis, raw cells and LZRS virus-infected cells were co-cultured for 24 h and 72 h prior to flow cytometric analysis. BD LSRFortessa cell analyzer (BD Biosciences) and BD FACSDiva™ software (BD Biosciences) were used for flow cytometry and analysis of cell distribution, respectively.

### Transcriptome analysis and pathway modeling

Total RNA was extracted from empty vector control and *FCN3*-virus-transduced A549 cells, and mRNA libraries were constructed by the TruSeq Stranded mRNA Preparation kit (Illumina) according to the manufacturer’s instructions. RNA-seq was performed with Illumina NovaSeq 6000 sequencing platform for 101-mer paired-end reads (DNA Link Inc., Seoul, Korea). Sequencing reads were aligned to the reference human genome (hg19) using STAR alignment program (version 2.6.1d)^22^ after trimming adapter sequences and discarding low-quality reads using Fastx_toolkit (version 0.0.13). Transcriptome abundance was estimated with RSEM (version 1.3.3)^16^. DEGs were identified using DESeq2^23^ with the FDR threshold of 0.01. Gene set analyses for DEGs were performed with EnrichR^24^ using KEGG pathways and gene ontology (GO) terms. The RNA-seq data have been deposited in the Gene Express Omnibus (GEO) database [GEO: GSE160946].

### Xenograft tumor model

A total of 2 × 10^6^ A549 cells in 100 μL of PBS were subcutaneously injected into the back of 5 week old female BALB/c nude mice (Orient Bio Inc., Seongnam, Korea). Weight of the mice and size of the tumors were measured every 5 days. The tumor volume was calculated as 0.5 × length × width^2^. The mice were sacrificed and tumor tissues were excised and weighted.

## Results

### Down-regulation of *FCN3* in LUAD tissues

**Fig. 1:**
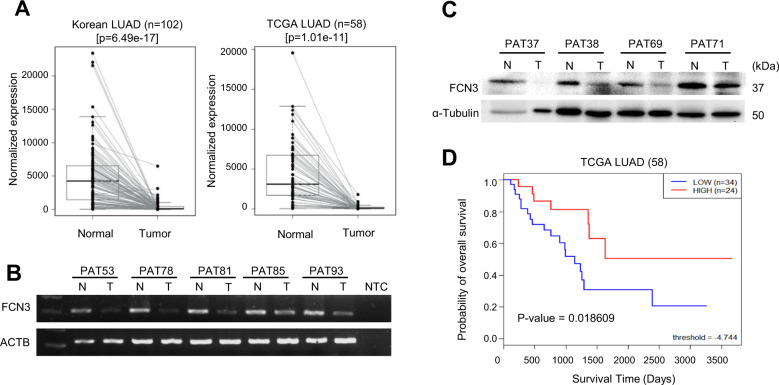
*FCN3* is down-regulated in LUAD. **A** Box-plots of *FCN3* RNA expression in normal-tumor matched 102 LUAD patients in this study (left panel) and of normal-tumor matched 58 patients from TCGA data (right panel). The bold line in the middle of each box represents the median level. **B** Conventional RT-PCR confirmation using normal (N) and tumor (T) tissue samples from 5 representative LUAD patients. ACTB was used as an endogenous reference gene. Note that mRNA expression of *FCN3* is markedly down-regulated in tumor tissues. NTC stands for no template control. **C** Immunoblot analysis of FCN3 expression in normal (N) and tumor (T) tissue samples of 4 representative patients. ɑ–Tubulin was used as the loading control. FCN3 down-regulation is confirmed. **D** Kaplan–Meier survival curves of 58 TCGA LUAD patients divided into two groups based on *FCN3* expression. Long-term survival was seen to be significantly correlated with high expression of *FCN3*.

We have previously reported generating RNA-seq data from paired tumor and normal LUAD samples of over 100 never-smoker female patients^25^ (a comprehensive report on somatic mutations and DEGs will be made elsewhere). Among the DEGs down-regulated in tumor tissues, which represent potential tumor suppressors, was *FCN3*, a gene encoding a lectin protein proposed to be involved in innate immunity. *FCN3* showed a consistency >95% (i.e. 97 out of 102 patients showed down-regulation in tumor tissues) and the average Log_2_ fold change of 3.72 (Fig. 1A). Similar results were obtained from normal-tumor matched samples of 58 patients registered in TCGA (Fig. 1A). We have examined a subset of patient samples by RT-PCR (Fig. 1B) and by immunoblotting (Fig. 1C) and confirmed the results from RNA-seq. We proceeded to examine if the expression level of *FCN3* can be a prognostic indicator for life expectancy after diagnosis. Indeed, for the 58 TCGA LUAD cohorts, higher expression levels of *FCN3* were strongly associated with better survival consistent with a meaningful role of *FCN3* as a tumor suppressor gene (Fig. 1D). We also examined the expression pattern of *FCN3* in other tumors with a sufficient number of matched normal and tumor tissues. At least five out of 12 types of cancer examined including breast invasive carcinoma (BRCA), kidney renal papillary cell carcinoma (KIRP), liver hepatocellular carcinoma (LIHC), lung squamous cell carcinoma (LUSC) and LUAD show significant down-regulation of *FCN3* in tumor tissues (Supplementary Fig. 1A). The degrees of down-regulation are particularly significant for LIHC, LUAD, and LUSC (Supplementary Fig. 1B) which are the cancers of tissues where *FCN3* is highly expressed^8^ (also see “Discussion”).

### *FCN3* induces cell cycle arrest and apoptosis of LUAD cells

**Fig. 2:**
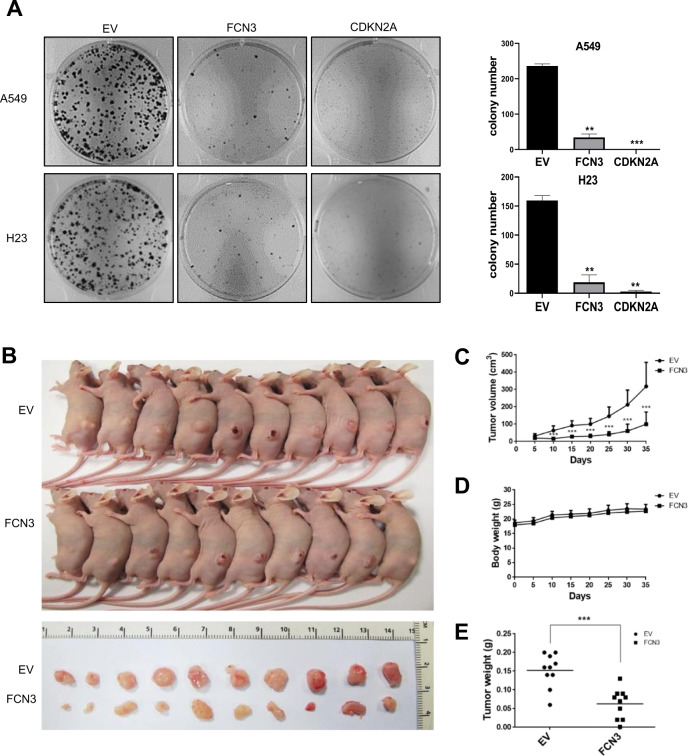
Cell growth is inhibited by *FCN3*. **A** A549 and H23 cells were transduced with control empty virus (EV), FCN3-expressing virus, or CDKN2A-expressing virus and allowed to grow for 8 and 12 days, respectively. Resulting colonies were stained with Coomassie blue, and the number was determined. Graphs to the right show result from three independent experiments as mean ± SEM. Note the strong decrease in the number of colonies in *FCN3*-expressing cells. **B** Photographs of mice with xenograft tumors (top) and of excised tumors (bottom). The mice were sacrificed 35 days after cell injection. Note that tumors from the FCN3-expressing A549-injected group show decreased tumor development compared to mice in the control group injected with control EV-transduced A549 cells. **C** Growth curves of xenograft tumor. Tumor volume was measured every 5 days. *N* = 10 for both groups. **D** Body weight of BALB/c nude mice was measured every 5 days. **E** The weight of all tumors isolated from EV group and *FCN3* group. The lines in the middle represent the median levels. (**) and (***) represent *P*-values of <0.01 and <0.001 from *t* tests, respectively.

We sought to determine if FCN3 negatively affects cell growth using colony formation assay and BrdU labeling assay. Two independent LUAD cell lines, A549 and H23 were targeted for ectopic expression of FCN3 (Supplementary Fig. 2A). Formation of colony was dramatically inhibited upon expression of *FCN3* in a similar manner to that of *CDKN2A*, a well-established tumor suppressor gene (Fig. 2A). Consistently, proportions of BrdU positive cells were significantly lower among FCN3-expressing cells (Supplementary Fig. 2B). These results clearly indicated that *FCN3* can inhibit cell proliferation. We also examined the tumor suppressor activity of *FCN3* using a xenograft model. A549 cells were transduced either with control virus or FCN3-expressing virus and transplanted to NSG mice. Over the course of 5 weeks, changes in tumor volumes were tracked. A clear and significant difference was observed between control and *FCN3* groups consistent with inhibition of cell growth by *FCN3* (Fig. 2B–E).

**Fig. 3:**
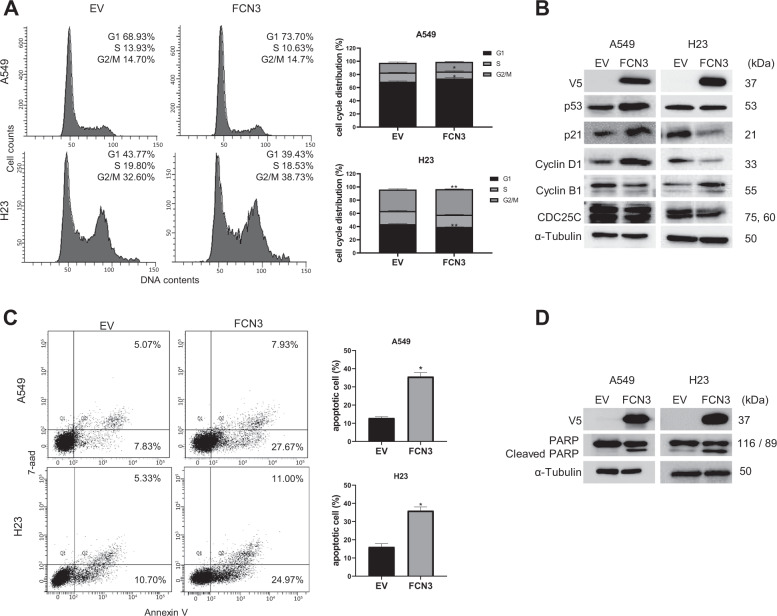
Ectopic expression of *FCN3* induces cell cycle arrests and apoptosis. **A** A549 and H23 cells were infected with control virus (EV) or *FCN3*-expressing virus, and cell cycle progression was analyzed by flow cytometry 72 h after infection. Representative histograms are shown, and proportions of cell cycle distribution were presented as bar graphs. G1 arrest was observed in A549 cells, while G2/M arrest occurred in H23 cells. Data are mean ± SEM of three independent experiments. **B** Immunoblots of various cell cycle markers in A549 and H23 cells 72 h after viral transduction. ɑ–Tubulin was used as the loading control. **C** A549 and H23 cells were transduced with control virus (EV) or *FCN3*-expressing virus, and apoptotic cells were analyzed with flow cytometry 96 h after infection. Proportions of apoptotic cells are presented as bar graph. The percentage of apoptotic cells is increased with FCN3 over-expressed cells compared to that in control. Data summarized in the graph are mean ± SEM of three independent experiments. **D** Immunoblots showing PARP in A549 and H23 cells after 72 h of viral infection. Cleaved PARP is increased in *FCN3*-expressing cells. (*) and (**) represent *P*-values of <0.05 and <0.01 from *t* tests, respectively.

Tumor suppressors often induce cell cycle arrest and apoptosis. *FCN3* expression on A549 and H23 cells led to G1 and G2/M arrest, respectively, and showed consistent changes in levels of cell cycle protein markers (Fig. 3A, B). Specifically, in A549 cells p53, p21, and Cyclin D1 were up-regulated while cyclin B1 and CDC25C were down-regulated (Fig. 3B). In contrast, in H23 cells P21 and Cyclin D1 were down-regulated while Cyclin B1was noticeably up-regulated. The difference may result from the status of the p53 gene which is mutated in H23 cells^26,27^. It has been reported that lack of wild-type p53 protein can lead to bypassing of G1 checkpoint and to G2/M arrest phenotype instead^28–30^. Delayed cell cycle upon FCN3 expression appears to ultimately lead to apoptosis as can be seen from increased proportions of Annexin V positive cells in both A549 and H23 cells (Fig. 3C). This was corroborated by the enhanced appearance of cleaved PARP upon FCN3 expression (Fig. 3D).

### Intracellular mechanism for tumor suppressor activity of *FCN3*

**Fig. 4:**
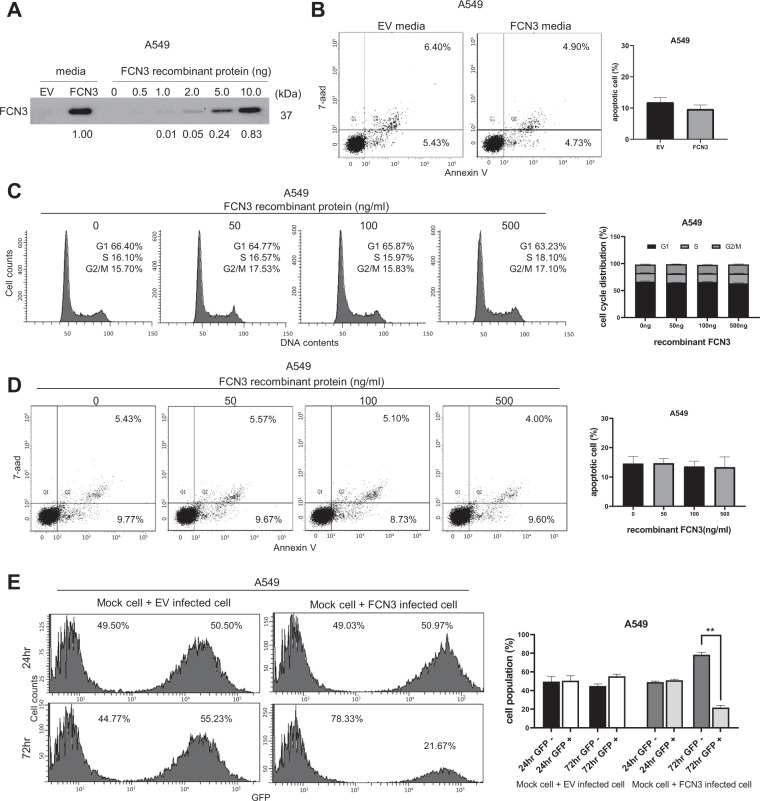
Tumor suppressor activity of *FCN3* involves an intracellular mechanism. **A** Immunoblot showing the secreted FCN3 in cultured media of *FCN3*-virus-transduced A549 cells but not in culture media of control virus (EV)-transduced cells. Indicated amounts of recombinant FCN3 protein are also shown. **B** Culture media from control virus- or *FCN3*-virus-transduced cells were applied to A549 cells, and apoptosis was analyzed by flow cytometry after 96 h. Data summarized in the graph are mean ± SEM of three independent experiments. Note no significant difference was seen. **C** Cell cycle analyses of A549 cells by flow cytometry 72 h after application of indicated doses recombinant FCN3. No alteration in cell cycle progression was observed. Data are mean ± SEM of three independent experiments. **D** Apoptosis was examined 96 h after application of indicated doses recombinant FCN3. No alteration in proportions of apoptotic cells was noticed. Data are mean ± SEM of three independent experiments. **E** A549 cells with or without viral transduction were mixed and co-cultured for 24 h and 72 h. Proportions of GFP-negative and -positive cell populations were analyzed by flow cytometry. After 72 h, GFP-positive proportion was decreased only in the case of *FCN3*-virus-transduced cells. Data are mean ± SEM of three independent experiments, and (**) represents *P*-value of <0.01 from *t* test.

Consistent with the presence in serum and activation of the complement pathway, secreted FCN3 protein was readily detected in cell culture media after ectopic expression (Fig. 4A; Supplementary Fig. 3A). Comparison with varying concentrations of recombinant FCN3 indicated that approximately 200 ng/ml of FCN3 was present. Surprisingly, culture media from *FCN3*-expressing cells did not induce additional cell death as measured by flow cytometry (Fig. 4B; Supplementary Fig. 3B). Also, the addition of recombinant FCN3 even at levels comparable or higher than that secreted from cells failed to induce cell cycle arrest or apoptosis (Fig. 4C, D; Supplementary Fig. 3C, D). We mixed control cells without viral transduction with either control virus-transduced cells or *FCN3*-virus-transduced cells and examined the changes in cellular proportions taking advantage of GFP expression from the recombinant viruses. Consistent with results described above, *FCN3*-expressing cells but not control virus–transduced cells showed a significant decrease in proportions in time (Fig. 4E; Supplementary Fig. 3E). Taken together, these results strongly suggest that tumor suppressor activity of *FCN3* is cell-autonomous and mediated by an intracellular mechanism.

**Fig. 5:**
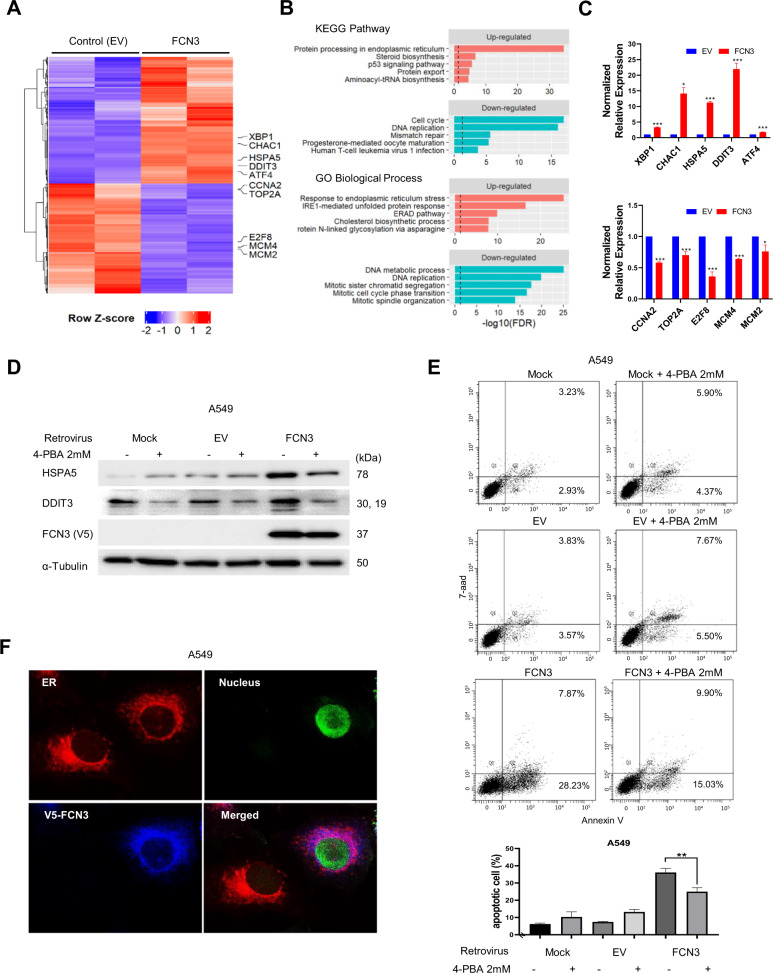
Transcriptomic analyses indicate *FCN3* induces ER stress. **A** Expression heatmap view using 1,055 differentially expressed genes (DEGs) from duplicate samples of control empty virus (EV) and *FCN3*-virus-transduced cells. Genes with indicated gene symbols were used in validation experiment. **B** Gene set analysis on 595 up-regulated genes (top) and 460 down-regulated genes (bottom) for KEGG pathways and Gene Ontology (GO) biological processes. Top 5 scoring terms are shown in the plots. The full list is provided in Supplementary Table 3. Black dashed vertical lines represent the FDR threshold of 0.05. **C** Quantitative real time RT-PCR was performed to confirm RNA-Seq results. Five up-regulated and five down-regulated genes were examined after transduction with control or *FCN3*-expressing virus. **D** Immunoblots of ER stress markers HSPA5 and DDIT3 in A549 cells with or without 4-phenylbutyric acid (4-PBA) treatment. A549 cells were mock treated, EV transduced or *FCN3*-virus-transduced and cultured for 48 h prior to sampling. HSPA5 and DDIT3 were induced in *FCN3*-virus-transduced cells, and 4-PBA treatment effectively down-regulated these ER stress markers. ɑ–Tubulin was used as the loading control. **E** Apoptosis of A549 cells were examined by flow cytometry 96 h after viral transduction with or without 4-PBA. The percentage of apoptotic cells decreased with the alleviation of ER stress by 4-PBA treatment. Graph to the bottom summarizes the results. Data are mean ± SEM of three independent experiments, and (**) represents *P*-value of <0.01 from *t* test. **F** Subcellular localization of FCN3 in A549 cells. Nuclear GFP expression indicates the *FCN3*-virus-transduced cell. V5 staining shows localization of ectopic FCN3 in ER.

In order to dissect the molecular nature of the tumor suppressor activity, we carried out transcriptome sequencing for *FCN3*-expressing A549 cells and control samples in duplicates. Our computational pipelines identified 1055 DEGs with FDR < 0.01 (Fig. 5A and Supplementary Table 2). We proceeded to carry out a functional enrichment analysis for 595 up-regulated and 460 down-regulated genes separately using EnrichR web application for the KEGG pathways and gene ontology (GO) terms of biological processes (Supplementary Table 3). Up-regulated genes were enriched with several terms associated with endoplasmic reticulum (ER) functions including “protein processing in ER”, “response to ER stress” and “IRE1-mediated unfolded protein response (UPR)” (Fig. 5B). This strongly suggests that ectopic expression of FCN3 caused ER stress which in turn activated the UPR process. Down-regulated genes were enriched with terms on cell cycles and DNA replication, consistent with diminished cellular proliferation. RT-PCR analyses on multiple up-and down-regulated genes provided consistent results (Fig. 5C). Importantly, among the up-regulated were *HSPA5* (also known as *GRP78*) and *DDIT3* (also known as *CHOP*), two of the hallmark genes of ER stress-induced UPR activation^31,32^. We confirmed the induction of the two genes by ectopic expression of *FCN3* at the protein level as well (Fig. 5D; Supplementary Fig. 4A). Importantly, alleviation of ER stress with 4-PBA treatment also reduced *FCN3*-induced expression of HSPA5 and DDIT3 (Fig. 5D; Supplementary Fig. 4A) as well as apoptosis (Fig. 5E; Supplementary Fig. 4B). We also confirmed that FCN3 was localized to ER as expected for a protein which is ultimately secreted (Fig. 5F).

### Fibrinogen-like domain is necessary and sufficient for tumor suppressor activity

**Fig. 6:**
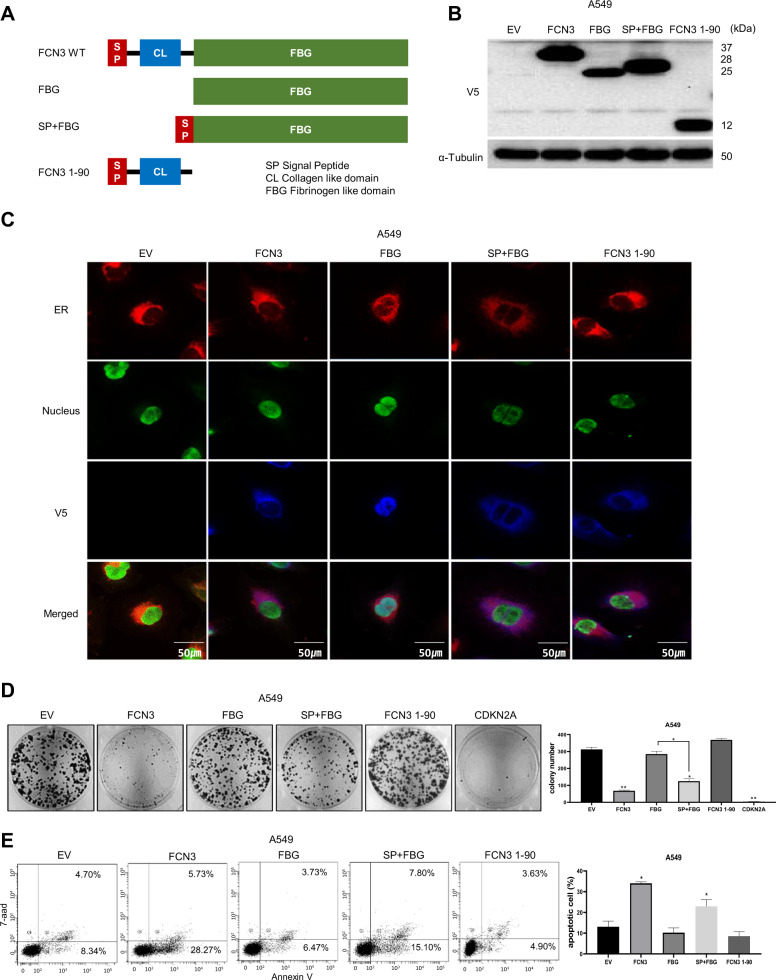
The FBG domain localized to ER is necessary and sufficient for tumor suppressor activity of *FCN3.* **A** Schematic illustration of various *FCN3* derivatives: wild type *FCN3*, FBG domain, SP (signal peptide) + FBG domain, and *FCN3* 1-90. **B** Immunoblots showing ectopic expression of various FCN3 derivatives in A549 cells. Antibody against V5 epitope was used. ɑ–Tubulin was used as the loading control. **C** Expression and subcellular localization of FCN3 derivatives in A549 cells. Nuclear GFP expression indicates the virus-transduced cell. V5 staining shows localization of various FCN3 derivatives. Note no V5 staining in control virus (EV)-transduced cells and nuclear localization of FBG. **D** Colony formation assay after transducing with control virus or viruses expressing the indicated FCN3 derivatives in A549 cells. Note SP + FBG has growth inhibition effect. Numbers of colonies were counted and presented in bar graphs. Data are mean ± SEM of three independent experiments. **E** Apoptosis of A549 cells was evaluated with flow cytometry after transducing with control virus or viruses expressing the indicated FCN3 derivatives. (*) and (**) represent *P*-values of <0.05 and <0.01 from *t* tests, respectively.

To further understand ER stress-mediated by *FCN3*, we generated several *FCN3* derivatives (Fig. 6A) and tested their tumor suppressor activity using colony formation assay and flow cytometry for apoptosis. The proteins of expected sizes were generated in both A549 and H23 cells (Fig. 6B; Supplementary Fig. 5A). Cellular localization of each derivative was also determined (Fig. 6C). A deletion derivative containing just the C-terminal fibrinogen-like domain (FBG) was found in the nucleus unlike the others containing the signal peptide for secretion. This derivative failed to induce growth retardation or cell death (Fig. 6D, E). A derivative containing just the N-terminal collagen-like domain (*FCN3* 1-90) was also not able to bring growth inhibition or cell death even though it localized to ER and the amount of protein was comparable to the wild type *FCN3* (Fig. 6B–E). Of note, upon inclusion of the signal sequence, the fibrinogen-containing derivative protein (SP + FBG) localized to ER, inhibited cell growth and induced cell death (Fig. 6C–E). We saw similar effects of expressing these derivatives in H23 cells as well (Supplementary Fig. 5B, C). Interestingly, when we examined secretion of FCN3 derivatives in cell cultured media by immunoblotting, only the wild type *FCN3* and *FCN3* 1-90 derivative were detected in culture media (Supplementary Fig. 5D). Therefore, SP + FBG must have induced cell death via an intracellular mechanism without being secreted into the media. Taken together, the data strongly indicate that ER stress induced by FCN3 is not a side effect of non-specific protein overload in ER and that the apoptosis is not caused by already known activity of FCN3 such as complement activation. Rather, a specific signaling mechanism mediated by fibrinogen-like domain within ER appears to be in operation.

## Discussion

Since its identification and cloning as the gene encoding the Hakata Antigen, *FCN3* has been characterized for its role in innate immunity which is mediated by activating the lectin complement pathway^5–7^. Another activity of FCN3 reported has to do with the clearance of apoptotic cells suggesting involvement in tissue homeostasis and possibly a protective role against the development of autoimmunity^11^. As noted above, virtually no association has been made between *FCN3* and LUAD carcinogenesis. One exception reported by Michalski and coworkers describes recombinant *FCN3* binding strongly to cells from multiple ovarian cancer cell lines^33^. This activity was apparently inhibited by carbohydrate ligands indicating mediation by the fibrinogen-like domain of FCN3. They further reported that FCN3 preferentially binds to ovarian tissue sections of patients with malignant tumors in comparison to tissue sections of patients without. Taken together, a possible function in immune response targeting malignant ovarian cancers was proposed, but no further examinations for substantiation of this hypothesis have followed.

Our mechanistic analyses indicate that at least for LUAD it is not likely to be the activation of immune response that is responsible for the tumor suppressor activity of *FCN3*. Clearly, *FCN3*-induced cell death took place in a cell-autonomous manner: secreted or recombinant version had no effect on LUAD cells. We show that tumor suppressor activity of *FCN3* is based on induction of ER stress response which can result in cell death under certain circumstances. Interestingly, the fibrinogen-like domain, when localized to ER, is both necessary and sufficient for induction of apoptosis suggesting a lectin-like signaling activity targeting a glycosylated protein located within ER is possibly involved.

Surprising though it may be, there is an interesting precedent for induction of ER stress mediated by an otherwise secreted protein. Block and coworkers reported that complement factor properdin (*CFP*), known to be a secreted protein and a regulator of innate response functioning as a pattern recognition molecule, suppresses the growth of breast cancer cells by inducing ER stress rather than by eliciting immune responses^34^. It is interesting that CFP and FCN3 are both secreted proteins involved in innate immunity functioning through activation of complement pathway although the pathways they activate are distinct. Whether co-option of these genes for tumor suppressors has common mechanistic or evolutionary aspect would be an interesting topic for future investigation.

Our analyses of *FCN3* expression in LUAD patients indicate that the gene could be a potential diagnostic marker. A highly consistent (>95%) down-regulation in tumor tissues compared to matched normal tissue was seen in both our cohorts and in TCGA patient groups. Furthermore, *FCN3* may have prognostic value as statistically significant difference in survival expectancy was seen between *FCN3*-high expression group and *FCN3*-low expression group although the cohort size is somewhat limited. Of interest, *FCN3* does not appear to be a universal tumor suppressor in that aside from LUAD we saw statistically significant down-regulation only in BRCA, KIRP, LIHC, and LUSC. It is noteworthy that aside from lung, liver is another tissue in which *FCN3* has been reported to be strongly expressed^8^. Also, at least RNA-level analyses indicate relatively high expression levels in the kidney and breast (see https://www.genecards.org/cgi-bin/carddisp.pl?gene=FCN3). Clearly, studies using cancer cells derived from these cancer types would be necessary to conclude if *FCN3* functions as tumor suppressor and if a common mechanism involving the induction of ER stress is at work.

## Supplementary information

Supplementary Figures and Table 1

Supplementary Table 2

Supplementary Table 3

Legends for Supplementary Data
