# Supplementary material for: FCN3 functions as a tumor suppressor of lung adenocarcinoma through induction of endoplasmic reticulum stress
Source: Cell Death Dis. 2021 Apr 15;12(4):407. doi: 10.1038/s41419-021-03675-y (PMC8050313; doi:10.1038/s41419-021-03675-y)
Supplement: Supplementary file 1 — Supplementary Figures and Table 1 [file 41419_2021_3675_MOESM1_ESM.pdf]

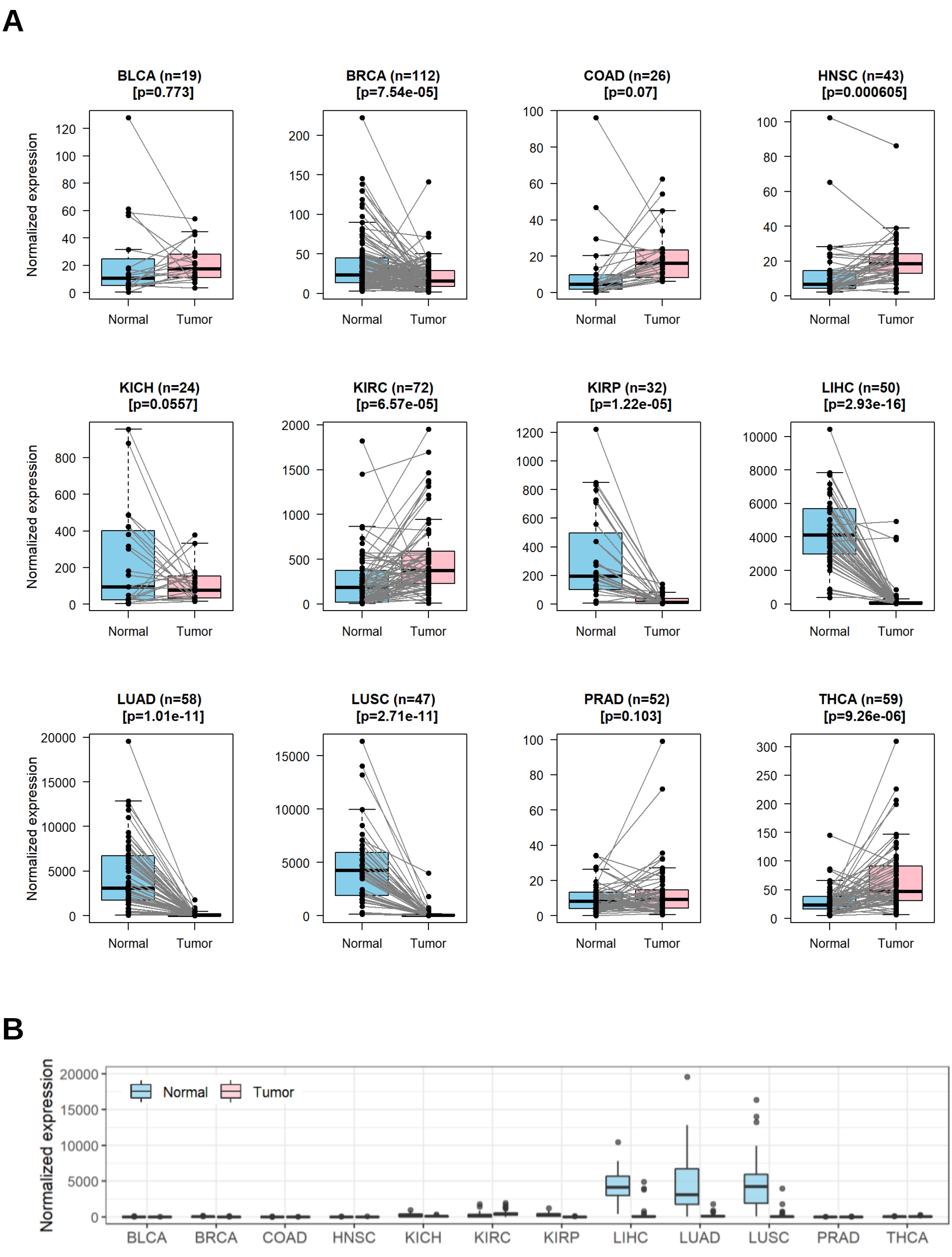

Supplementary Fig. 1. Expression analysis of *FCN3* using normal-tumor matched patient data from TCGA.

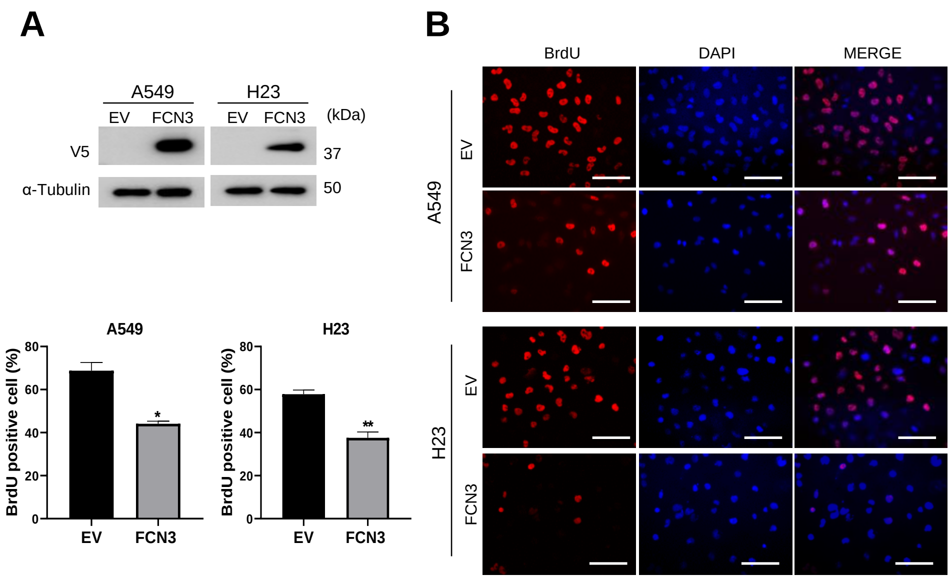

Supplementary Fig. 2. Expression of FCN3 and BrdU labeling.

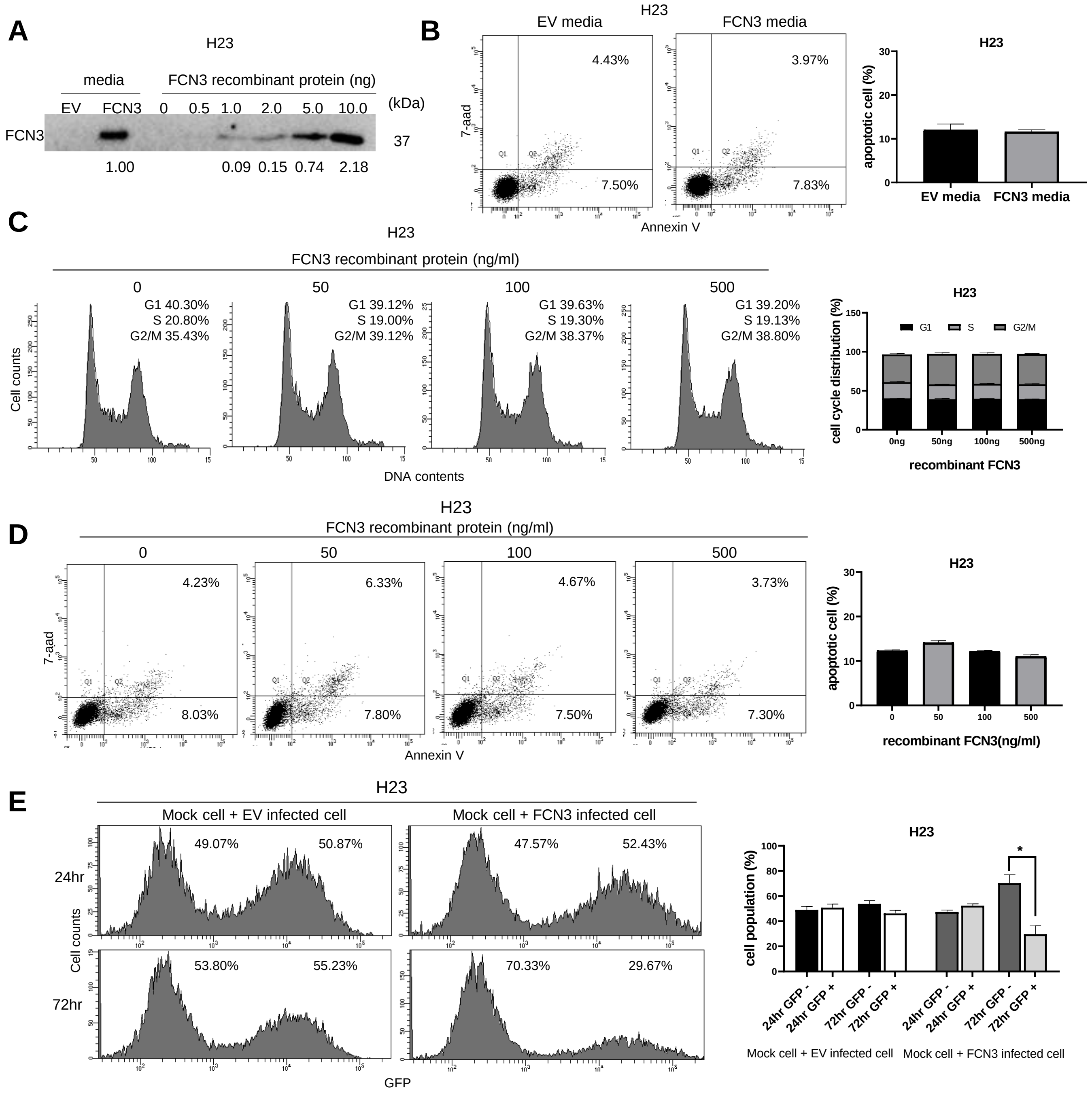

Supplementary Fig. 3. Tumor suppressor activity of FCN3 involves an intracellular mechanism.

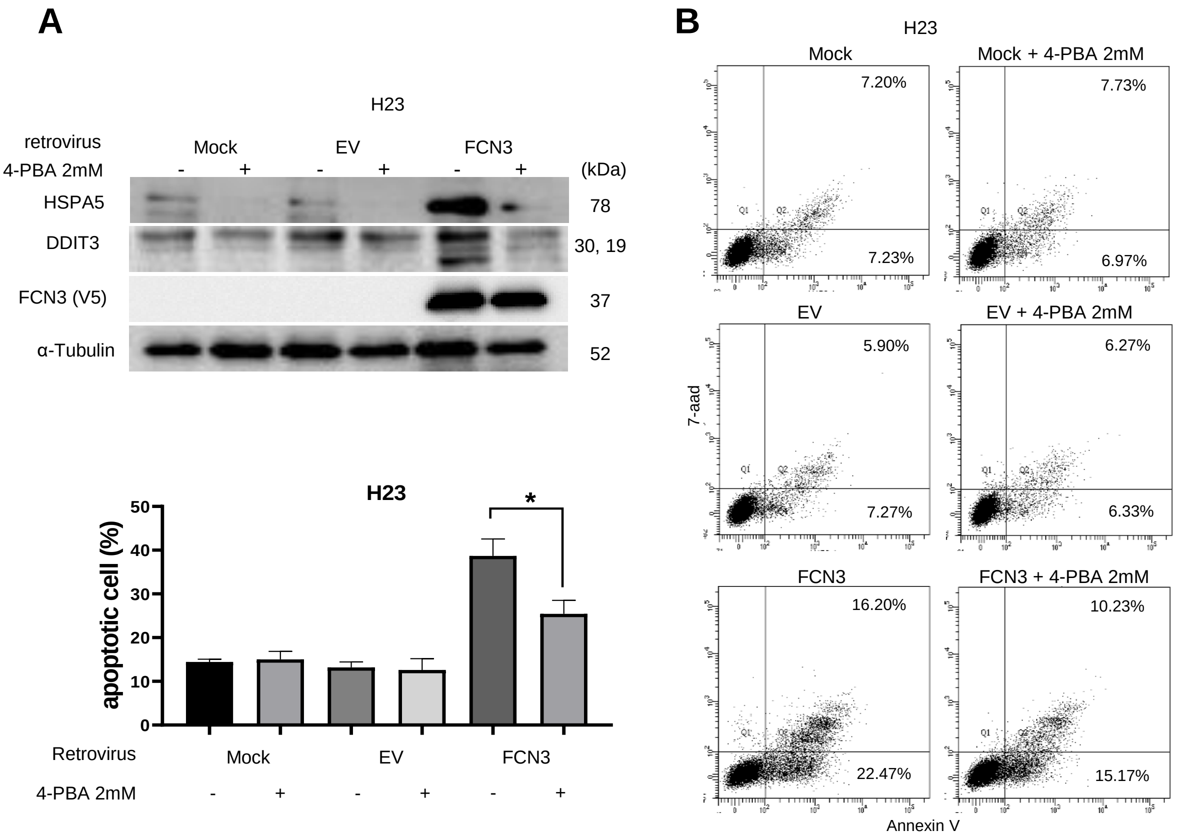

Supplementary Fig. 4. FCN3 induces ER stress in H23 cells.

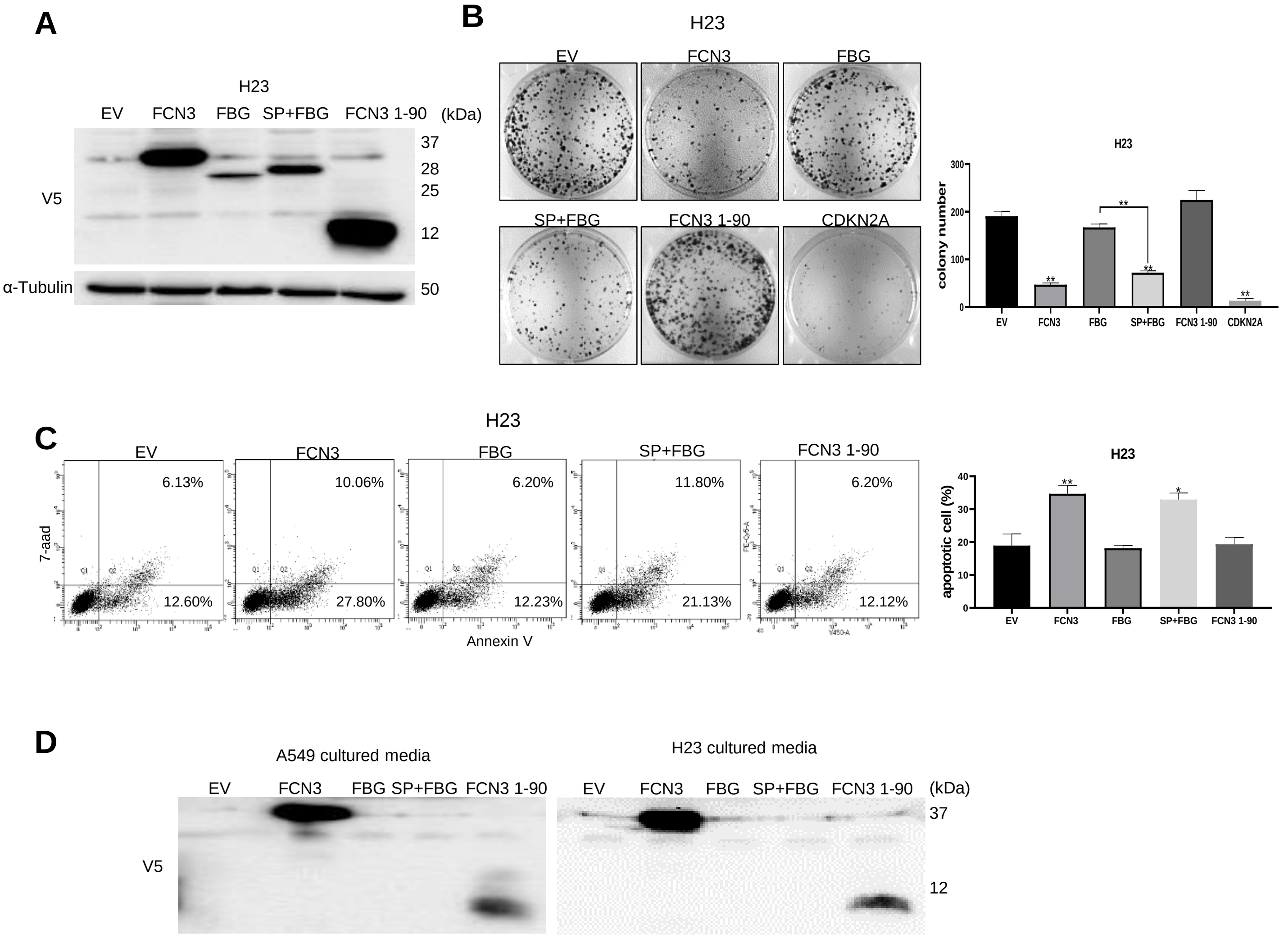

Supplementary Fig. 5. Tumor suppressor activity of FCN3 derivatives in H23 cells.

Gene	Oligonucleotide sequence (5’→3’)	
**FCN3**	Forward	CCCAGTCTTTTGTGACATGGA
	Reverse	CCTGCTCTGTAGGAGGACCA
**ATF6**	Forward	AGTTCGTCAGTTCCTCCTTACCTC
	Reverse	AGTTCTCTGCCTGCCACCAAG
**CCNA2**	Forward	TGCATCTCTGGGCGTCTTTG
	Reverse	ACCCGGCCAAAGAATAGTCG
**CHAC1**	Forward	TGGTGACGCTCCTTGAAGATC
	Reverse	GCACTGCCTCTCGCACATT
**DDIT3**	Forward	GCTCAGGAGGAAGAGGAGGA
	Reverse	TCCTGCTTGAGCCGTTCATT
**E2F8**	Forward	GGACAGTACCTGCTTGCCTT
	Reverse	GGAGGTCGCTTGACAGGAAA
**HSPA5**	Forward	CACTCCTGAAGGGGAACGTC
	Reverse	ACCACCTTGAACGGCAAGAA
**MCM2**	Forward	AATTTCGTCCTGGGTCCTTT
	Reverse	CACTTTGCCTGGACTCTCCT
**MCM4**	Forward	TTCTTTGACCGTTACCCTGA
	Reverse	ACACTTGGCACTGGAAGAAG
**TOP2A**	Forward	GCTGGATCAGTGGCTGAAAT
	Reverse	AATGGGCTGCAAGAGGTTTA
**XBP1**	Forward	TTGCTGAAGAGGAGGCGGAAG
	Reverse	GGTCCAAGTTGTCCAGAATGC
**ACTB**	Forward	ACAGAGCCTCGCCTTTGC
	Reverse	GAAGCCGGCCTTGCACAT
**HPRT1**	Forward	ACACTGGCAAAACAATGCAG
	Reverse	GTGGGGTCCTTTTCACCAG

Supplementary Table 1. Oligonucleotide primers used in RT-PCR and quantitative real time RT-PCR.
