# Supplementary material for: FCN3 functions as a tumor suppressor of lung adenocarcinoma through induction of endoplasmic reticulum stress
Source: Cell Death Dis. 2021 Apr 15;12(4):407. doi: 10.1038/s41419-021-03675-y (PMC8050313; doi:10.1038/s41419-021-03675-y)
Supplement: Supplementary file 4 — Legends for Supplementary Data [file 41419_2021_3675_MOESM4_ESM.docx]

**Legends for Supplementary Data**

**Supplementary Fig. 1. Expression analysis of *FCN3* using normal-tumor matched patient data from TCGA.** (A) Box-plots of *FCN3* RNA expression in 12 types of cancer with at least > 19 matched normal-tumor patient data. Bold line in the middle of each box represents the median value. Abbreviations: BLCA, Bladder Urothelial Carcinoma; BRCA, Breast invasive carcinoma; COAD, Colon adenocarcinoma; HNSC, Head and Neck squamous cell carcinoma; KICH, Kidney Chromophobe; KIRC, Kidney renal clear cell carcinoma; KIRP, Kidney renal papillary cell carcinoma; LIHC, Liver hepatocellular carcinoma; LUAD, Lung adenocarcinoma; LUSC, Lung squamous cell carcinoma; PRAD, Prostate adenocarcinoma; THCA, Thyroid carcinoma. (B) Box-plot of *FCN3* RNA expression of 12 types of cancer using a unified expression level scale. Note the high expression level in normal liver and lung tissues.

**Supplementary Fig. 2. Expression of FCN3 and BrdU labeling.** (A) Immunoblots with antibodies against V5 epitope showing ectopically expressed FCN3 in A549 and H23 cells. Empty virus (EV) is used as the negative control and ɑ–Tubulin was used as the loading control. (B) A549 and H23 cells were labeled with and stained for BrdU (red). DAPI staining (blue) indicates nuclei, scale bars: 100 ㎛ (400x). The proportions of BrdU positive cells were decreased in *FCN3*-expressing cells for both A549 and H23 cells. Four random visual fields were captured and cells were counted. Data are mean ± SEM of three independent experiments, and (*) and (**) represent *P*-values of <0.05 and <0.01 from *t*-tests respectively.

**Supplementary Fig. 3. Tumor suppressor activity of FCN3 involves an intracellular mechanism.** (A) Immunoblot showing the secreted FCN3 in cultured media of *FCN3* virus-transduced A549 cells but not in culture media of control virus (EV)-transduced cells. Indicated amounts of recombinant FCN3 protein are also shown. (B) Culture media from control virus- or *FCN3* virus-transduced cells were applied to H23 cells, and apoptosis was analyzed by flow cytometry after 96 hours. Data summarized in the graph are mean ± SEM of three independent experiments. Note no significant difference was seen. (C) Cell cycle analyses of H23 cells by flow cytometry 72 hours after application of indicated doses recombinant FCN3. No alteration in cell cycle progression was observed. Data are mean ± SEM of three independent experiments. (D) Apoptosis was examined 96 hours after application of indicated doses recombinant FCN3. No alteration in proportions of apoptotic cells was noticed. Data are mean ± SEM of three independent experiments. (E) H23 cells with or without virus transduction were mixed and co-cultured for 24 hours and 72 hours. Proportions of GFP-negative and -positive cell populations were analyzed by flow cytometry. After 72 hours, GFP-positive proportion was decreased only in the case of *FCN3* virus-transduced cells. Data are mean ± SEM of three independent experiments, and (*) represents *P*-value of <0.05 from *t*-test.

**Supplementary Fig. 4. FCN3 induces ER stress in H23 cells.** (A) Immunoblots of ER stress markers HSPA5 and DDIT3 in H23 cells with or without 4-phenylbutyric acid (4-PBA) treatment. H23 cells were mock treated, EV transduced or *FCN3* virus transduced and cultured for 48 hours prior to sampling. HSPA5 and DDIT3 were induced in *FCN3* virus-transduced cells, and 4-PBA treatment effectively down-regulated these ER stress markers. ɑ–Tubulin was used as the loading control. (B) Apoptosis of H23 cells were examined by flow cytometry 96 hours after viral transduction with or without 4-PBA. The percentage of apoptotic cells decreased with the alleviation of ER stress by 4-PBA treatment. Graph to the left summarizes the results. Data are mean ± SEM of three independent experiments, and (*) represents *P*-value of <0.05 from *t*-test.

**Supplementary Fig. 5. Tumor suppressor activity of FCN3 derivatives in H23 cells.** (A) Immunoblots showing ectopic expression of various *FCN3* derivatives in H23 cells. Antibody against V5 epitope was used. ɑ–Tubulin was used as the loading control. (B) Colony formation assay after transducing with control virus (EV) or viruses expressing the indicated *FCN3* derivatives in H23 cells. Note SP+FBG has growth inhibition effect. Numbers of colonies were counted and presented in bar graphs. Data are mean ± SEM of three independent experiments. (C) Apoptosis of H23 cells were evaluated with flow cytometry after transducing with control virus (EV) or viruses expressing the indicated *FCN3* derivatives. (*) and (**) represent *P*-values of <0.05 and <0.01 respectively. (D) Immunoblots showing *FCN3* derivatives secreted in culture media. Note for both A549 cells and H23 cells only the full length *FCN3* and *FCN3* 1-90 are found in the media.

**Supplementary Table 1.** Oligonucleotide primers used in RT-PCR and quantitative real time RT-PCR.

**Supplementary Table 2.** List of differentially expressed genes identified in *FCN3*-expressing cells. See Materials and methods and Figure 5.

**Supplementary Table 3.** The full list of terms from KEGG pathway and Gene Ontology enrichment analyses for differentially expressed genes.
